# Growth Mechanism and Properties of Self-Assembled InN Nanocolumns on Al Covered Si(111) Substrates by PA-MBE

**DOI:** 10.3390/ma12193203

**Published:** 2019-09-30

**Authors:** Y. L. Casallas-Moreno, S. Gallardo-Hernández, C. M. Yee-Rendón, M. Ramírez-López, A. Guillén-Cervantes, J. S. Arias-Cerón, J. Huerta-Ruelas, J. Santoyo-Salazar, J. G. Mendoza-Álvarez, M. López-López

**Affiliations:** 1 Instituto Politécnico Nacional, Unidad Profesional Interdisciplinaria en Ingeniería y Tecnologías Avanzadas, Av. IPN 2580, Gustavo A. Madero, Ciudad de México 07340, Mexico; 2 Departamento de Física, Centro de Investigación y de Estudios Avanzados del Instituto Politécnico Nacional, Apartado Postal 14-740, Ciudad de México 07360, Mexico; 3 Facultad de Ciencias Físico-Matemáticas, Universidad Autónoma de Sinaloa, Av. de las Américas y Blvd. Universitarios, Culiacán, Sinaloa 80000, Mexico; 4 Departamento de Ingeniería Eléctrica, Sección de Electrónica del Estado Sólido, Centro de Investigación y de Estudios Avanzados del Instituto Politécnico Nacional, Apartado Postal 14-740, Ciudad de México 07360, Mexico; 5 Centro de Investigación en Ciencia Aplicada y Tecnología Avanzada del Instituto Politécnico Nacional, Cerro Blanco 141, Querétaro C.P. 76090, Mexico

**Keywords:** InN nanocolumns, self-assembly of nanocolumns, molecular beam epitaxy, Al interlayer

## Abstract

Self-assembled InN nanocolumns were grown at low temperatures by plasma-assisted molecular beam epitaxy with a high crystalline quality. The self-assembling procedure was carried out on AlN/Al layers on Si(111) substrates avoiding the masking process. The Al interlayer on the Si(111) substrate prevented the formation of amorphous SiN. We found that the growth mechanism at $400{\;}^{\circ }C$ of InN nanocolumns started by a layer-layer (2D) nucleation, followed by the growth of 3D islands. This growth mechanism promoted the nanocolumn formation without strain. The nanocolumnar growth proceeded with cylindrical and conical shapes with heights between 250 and 380 nm. Detailed high-resolution transmission electron microscopy analysis showed that the InN nanocolumns have a hexagonal crystalline structure, free of dislocation and other defects. The analysis of the phonon modes also allowed us to identify the hexagonal structure of the nanocolumns. In addition, the photoluminescence spectrum showed an energy transition of 0.72eV at 20K for the InN nanocolumns, confirmed by photoreflectance spectroscopy.

## 1. Introduction

InN is a direct band-gap semiconductor with interesting physical properties such as; an infrared band-gap energy [1,2], a small electron effective mass [3,4], a high electronic mobility [5,6] and radiation resistance [7]. Important technological applications of this material include infrared light emitting diodes, high-speed and high-frequency devices, and photovoltaic systems [5]. To develop the above-mentioned devices, InN with a high crystalline quality is required. However, InN films commonly present a low crystal quality mainly caused by the low dissociation temperature and the lack of lattice-matched substrates [8]. These problems can be avoided with the growth of InN nanocolumns (InN NCs), where the lateral stress relaxation at sidewalls allows obtaining defect-free and strain-free nanostructures [9,10,11,12].

Thus far, most of the published work regarding InN growth is for temperatures higher than $400{\;}^{\circ }C$ [9,10,13,14]. For example, Kim et al. performed a study of InN growth at $500{\;}^{\circ }C$. At this temperature, the authors succeeded in growing 220-nm-thick InN epilayers on AlN/Si(111) substrates [15]. Kehagias et al. reported a 650 nm film in a two-step growth process, by first depositing a thin 20 nm InN layer on Si at low temperature ($225{\;}^{\circ }C$), prior to the main epilayer at $440{\;}^{\circ }C$ [16]. Anyebe et al. reported the evolution from InN nanorods to microstructure as a function of growth temperature above $490{\;}^{\circ }C$ on AlN/Si(111) [17], while Wang et al. studied the morphology of InN nanorods growth on GaN templates for temperatures in the range of 430–510 ${}^{\circ }C$ [18]. However, the use of low substrate temperatures is very important to avoid In interdiffusion and In droplet formation [19]. In this work, we report on the growth of InN NCs at a growth temperature of $400{\;}^{\circ }C$ by rf-plasma-assisted Molecular Beam Epitaxy (RF-MBE). We studied the nucleation mechanism of InN by reflection high-energy electron diffraction (RHEED). The nucleation process started layer by layer, followed by 3D nanocolumnar growth. The NCs structural and optical properties were studied by scanning electron microscopy (SEM), high-resolution transmission electron microscopy (HR-TEM), Raman spectroscopy, high-resolution x-ray diffraction (HR-XRD), photoluminescence and photoreflectance spectroscopy.

## 2. Materials and Methods

### 2.1. Samples Preparation

The growths were carried out in a Riber C21 MBE system equipped with a radio frequency (RF) nitrogen plasma source, and standard Knudsen cells. Prior to the growth of the InN NCs, the Si(111) substrate was chemically treated to remove the native silicon oxides and to form a thin fresh layer of oxides according to the procedure given in Ref. [20]. Then, the substrate was loaded into the growth chamber, where it was thermally cleaned at $900{\;}^{\circ }C$ for 10min. After this procedure, to avoid the degradation of the Si substrate by the direct exposition to N, a crystalline Al thin film of 30s was deposited over the substrate at a growth temperature (T${}_{g}$) of $850{\;}^{\circ }C$, employing an Al beam equivalent pressure (BEP${}_{Al}$) of $3.9\times 10^{-7}\;\mathrm{Torr}$. Subsequently, the N-plasma source was ignited at 150W with a N${}_{2}$ flow of 0.25sccm. Then, an AlN layer was formed by 6.5 min of unintentional nitridation of the Al layer with the nitrogen that leakages from the cell, even with the N-shutter is closed. The next step consisted of depositing an AlN layer for 30min at a temperature T${}_{g}$ of $850{\;}^{\circ }C$ with the two shutters open. Finally, for the growth of the InN, the temperature $T_{g}$ was lowered down to $400{\;}^{\circ }C$. To provide N-rich growth conditions appropriate for self-assembling of the nanocolumns, the N plasma source power was raised to 350W with a N${}_{2}$ flow of 0.75sccm, while the BEP${}_{In}$ was set to $2.0\times 10^{-7}\;\mathrm{Torr}$. The InN growth time was 1.5h. The growth stages were monitored in-situ by RHEED, with an acceleration voltage of 12kV.

### 2.2. Structural and Morphological Characterization

To evaluate the structural properties of the InN NCs, we employed HR-XRD by means of a MRD Xpert system from Panalytical (Panalytical, Malvern, Worcestershire, UK), which has a Cu α radiation in an open detector configuration. The surface morphology was analyzed by using a JEOL JSM-7401F SEM (JEOL, Akishima, Tokyo, Japan). The nanocolumns were additionally studied by HR-TEM images, obtained in a Jeol JEM 2010 (JEOL, Akishima, Tokyo, Japan) operating at 200kV, where NCs were peeled off from the substrates and mounted on holey carbon-coated copper grids. Micro-structural properties were studied by micro-Raman spectroscopy (NT-MDT, Integra Spectra, Zelenograd, Moscow, Russia) at room temperature in a backscattering configuration employing the 632nm line of a He–Ne laser (Thorlabs Inc, Newton, NJ, USA).

### 2.3. Optical Characterization

The optical properties were obtained by the photoluminescence technique at 20K using the 514.5nm line of an Ar+ laser as excitation source. The PL signal was focused on the entrance slit of the half-meter monochromator with a 600groove/mm grating and detected with a liquid-nitrogen-cooled InSb detector. For the photoreflectance measurements, the probe beam was produced by a 250W tungsten–halogen lamp coupled to 0.5m monochromator (SpectraPro-500i Acton Research Corporation, Acton, MA, USA). The reflectance was modulated with a 405nm laser diode and the spectra were collected by using a lock-in measurement with a Germanium (800–1800 nm) detector employing a 1050nm high pass filter (Thorlabs Inc., Newton, NJ, USA).

## 3. Results and Discussion

### 3.1. Structural and Morphological Characterization

**Figure 1:**
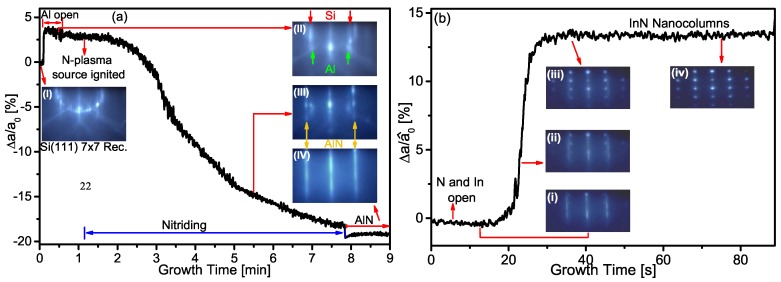
Variation of in-plane lattice constant during: (**a**) the growth of the crystalline Al thin film and AlN layer over the Si(111) substrate; and (**b**) InN nanocolumns.

The initial Al thin layer on the Si(111) substrate has two purposes: The first is to avoid the direct nitriding of the Si substrate, and therefore, the formation of amorphous SiN layer [9]. The second is to form an AlN layer by unintentional nitridation over which an AlN layer is grown with high crystalline quality. On this last layer, the InN has been grown. Each layer was monitored in situ by RHEED. The Si(111) 7×7 reconstruction after the thermal cleaning is shown in the RHEED Pattern (I) of Figure 1a. By depositing the Al layer over the Si-substrate, the 7×7 reconstruction began to disappear and a new diffraction spots appeared, as shown in Pattern (II). A step increase of the in-plane lattice constant difference $\left(\Delta a/a_{0}\right)$ was observed during the Al growth, as can be seen in Figure 1a; we noted that the lattice mismatch between the Al(111) and Si(111) substrate was about 4% [9], where $a_{0}$ is the lattice constant of Si(111). When the N-plasma was ignited, the formation of an AlN layer was clearly evidenced in the RHEED Pattern (III) with a 1×1 reconstruction, such as noted in Figure 1a. This AlN layer was formed by the bonding of the Al atoms present on the growth surface and the background N atoms that arrive at the surface, since the N and Al shutters are closed. The lattice constant difference $\left(\Delta a/a_{0}\right)$ decreased during the AlN layer formation until it reached the lattice mismatch of 18% with respect to the Si(111). This value does not correspond to a completely relaxed AlN, which has a lattice mismatch with Si of 19% [21]. Over this layer, the AlN growth continued (with both shutters Al and N open), where $\left(\Delta a/a_{0}\right)$ decreased rapidly until it reached the value of 19%, corresponding to a relaxed AlN, as is shown in Pattern (IV) of Figure 1a.

After the AlN layer, the growth of InN started under N-rich conditions, the N plasma source power and flow were raised with respect to the AlN layer, from 150 to 350W and 0.25 to 0.75sccm, respectively. When the InN began to nucleate, the RHEED pattern presented the same 1×1 reconstruction of the AlN and the $\left(\Delta a/{\hat{a}}_{0}\right)$ remained without changes respect to the AlN during 5s, suggesting the formation of a 2D growth or a wetting layer with a pseudomorphic growth, as shown in Pattern (i) of Figure 1b; here ${\hat{a}}_{0}$ is the lattice constant of AlN. Then, 3D spots began to appear in the 1×1 RHEED Pattern (ii), and the $\left(\Delta a/{\hat{a}}_{0}\right)$ increased rapidly. Finally, the diffraction pattern of the AlN disappeared and the spotty Patterns (iii)–(iv) distinctive of the nanocolumns were clearly observed. Likewise, $\left(\Delta a/{\hat{a}}_{0}\right)$ increased to 13.5% which is the lattice mismatch between InN and AlN [22], indicating that the InN is relaxed in the NCs, as noticed in Figure 1b. According to the results, the growth mechanism of the InN started by a layer–layer (2D) nucleation on AlN, followed by 3D islands, as a Stranski–Krastanov growth mode [23]. The 2D growth can be induced due to the high adhesion energy of the In and N atoms to the atoms of the AlN layer, and by the low growth temperature of $400{\;}^{\circ }C$. After the InN 2D growth, the 3D growth is generated by lattice relaxation under the N-rich condition [13,23,24]. In this case, the high density of the N atoms over the growth surface reduces the diffusion of the atoms on the InN thin layer, thus favoring the formation of 3D islands [25]. From these 3D islands, the InN NCs were growing. Thus, the NCs growth was free of seeding layers as the growth templates were fabricated by nanoimprint lithography [26]. In addition, it is important to point out that the NCs grew free of strain. Rocking curves revealed a FWHM value of $1.8{\;}^{\circ }$, caused by the mosaicity of the InN NC.

**Figure 2:**
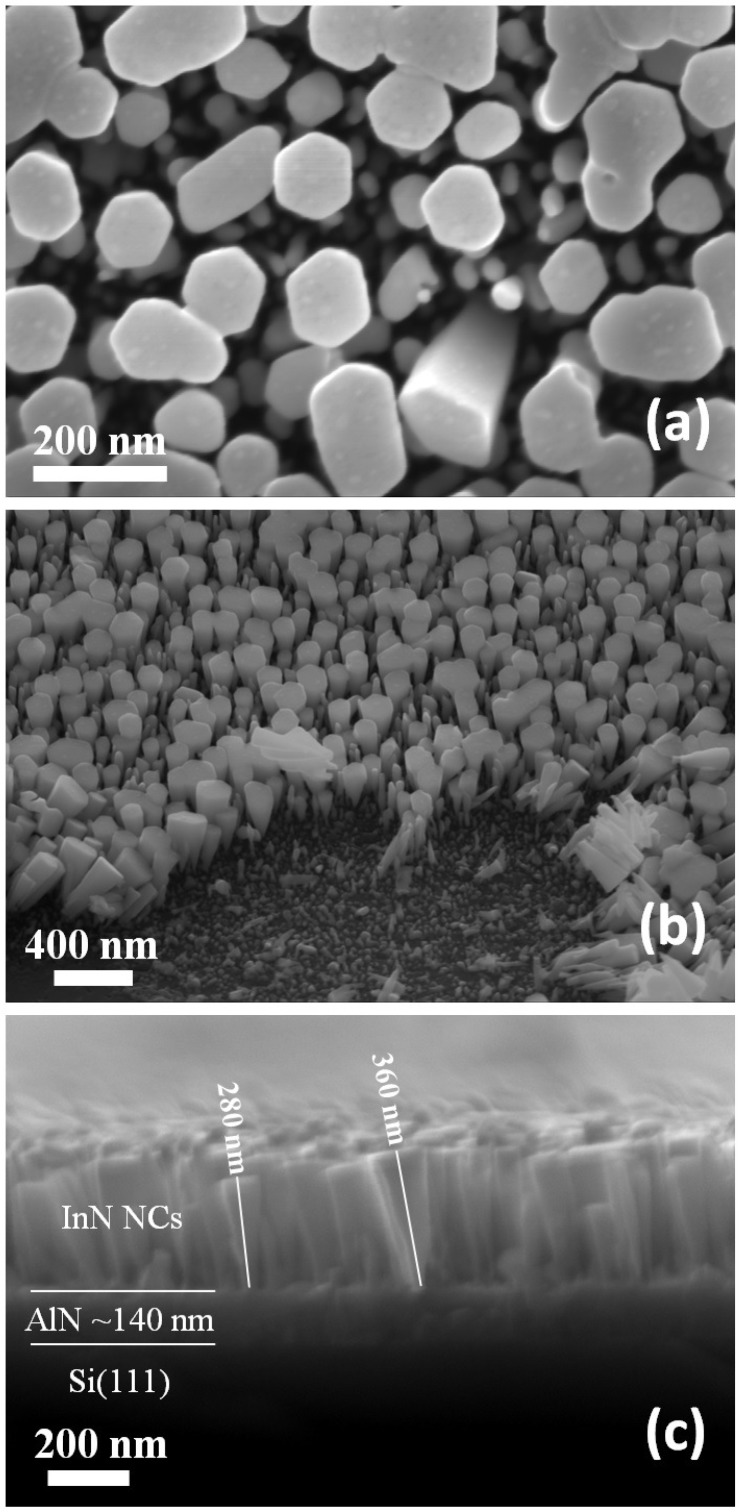
SEM (scanning electron microscope) micrographs of the InN nanocolumns: (**a**) top view; (**b**) lateral view at an angle of $45^{\circ }$; and (**c**) cross section view.

The surface morphology of the NCs was obtained by SEM measurements. Cylindrical and hexagonal NCs shapes were distinguished from top view micrographs shown in Figure 2a. The typical diameters were below 50nm for cylindrical and below 150nm for hexagonal NCs. The lateral view at an angle of $45^{\circ }$ in the SEM image shown in Figure 2b revealed that the hexagonal NCs have a conical shape, with an edge contact area on AlN layer smaller than the area seen on top view perspective. The area without NCs in SEM image corresponds to a region where the NCs were removed by the cantilever of an atomic force microscope. We also observed in this image some hexagonal coalesced nanocolumns on top due to the conical growth. From the lateral view micrographs presented in Figure 2c, the typical heights of the NCs were measured as 250–380 nm, and the approximated AlN layer thickness was 140nm.

**Figure 3:**
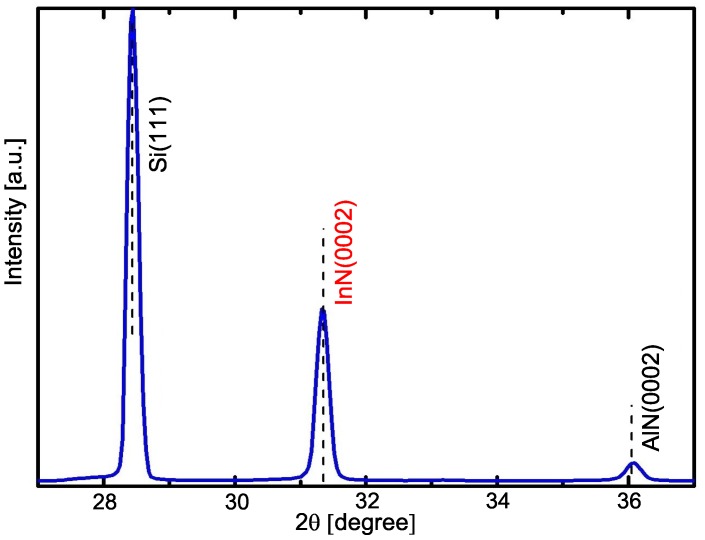
X-ray diffraction curve measured in θ–2θ configuration of the InN nanocolumns on Si(111) substrate.

Structural studies of the samples were carried out using XRD. A typical Bragg–Brentano diffraction measurement is shown in Figure 3. Three peaks were identified: the Si substrate peak coming from (111) planes, and two peaks from (0002) diffracting planes corresponding to the AlN and the InN. Dotted lines were positioned considering lattice parameters reported in the literature [14,21,22]. The peaks of the AlN layer and the InN NCs matched with the reported (dotted lines); therefore, we have NCs completely relaxed in hexagonal phase, which is consistent with the RHEED results.

**Figure 4:**
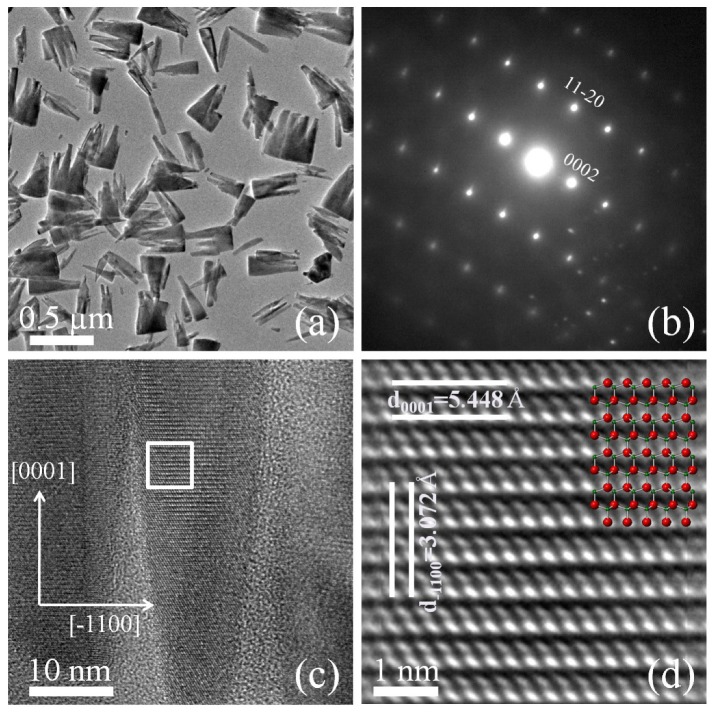
(**a**) Transmission electron micrograph of the InN nanocolumns peeled off the Si(111) substrate; (**b**) SAED pattern from a nanocolumn, where the direction of observation is [11$\bar{2}$0]; and (**c**) HR-TEM from an InN nanocolumn, where atomic planes can be appreciated, a square region marked in the picture is processed and displayed in (**d**).

The crystalline quality of the InN NCs was analyzed by HR-TEM. Figure 4a shows the shapes and sizes of the NCs. The selected area electron diffraction (SAED) pattern obtained from the NCs is shown in Figure 4b; the diffraction pattern presented a distribution corresponding to the crystalline hexagonal structure along the c-axis (the growth direction), which is in accordance with the results observed from XRD measurements. A nanocolumn image with conical shape on cross-sectional TEM is shown in Figure 4c, where the atomic arrangements of crystalline planes are seen. A detailed analysis was carried out in the search for crystalline defects, by measuring interplanar spacing and identifying crystallographic planes, employing Fourier and masking image treatments on the inset of Figure 4c, which is shown in Figure 4d. To identify the atom positions in the image, a ball-and-stick model of the InN wurtzite structure is overlapped. The model fits to the processed HR-TEM image if the nanocolumn is oriented along [0001] and [$\bar{1}$100] directions. Interplanar spacing were measured from the (0001) and ($\bar{1}$100) planes marked in Figure 4d for the nanocolumn; the lattice constant obtained from the HR-TEM analysis was $d_{\left(\bar{1}100\right)}=0.3072\;\mathrm{nm}$, which agrees well with the value calculated from interplanar spacing equation for a hexagonal lattice [27]. However, the lattice constant $d_{\left(0001\right)}=0.5448\;\mathrm{nm}$ is different from the accepted value of 0.57064nm [28]. This result could be explained assuming a slight tilt in depth of the nanocolumn around $17^{\circ }$ respect to the microscope electron beam. Such a tilt could be produced if the planes are on a crystalline facet. HR-TEM analysis on other InN NCs without facets, revealed that the spacing $d_{\left(0001\right)}$ is in accordance with the expected value. We want to remark that the NCs were free of structural defects or, at least, they were not observed in several explored regions. Additionally, there is no evidence of extended crystal defects in the InN NCs. This can be explained in terms of strain accommodation mechanisms at the interface, via lateral relaxation that allows strain relief. The result is consistent with reports regarding GaN [29] and InN NCs strain relaxation [9]. For example, Grandal et al. [9] showed that the lateral relaxation in the InN NCs grown by MBE is due to misfit dislocations periodically spaced at the InN/AlN interface.

**Figure 5:**
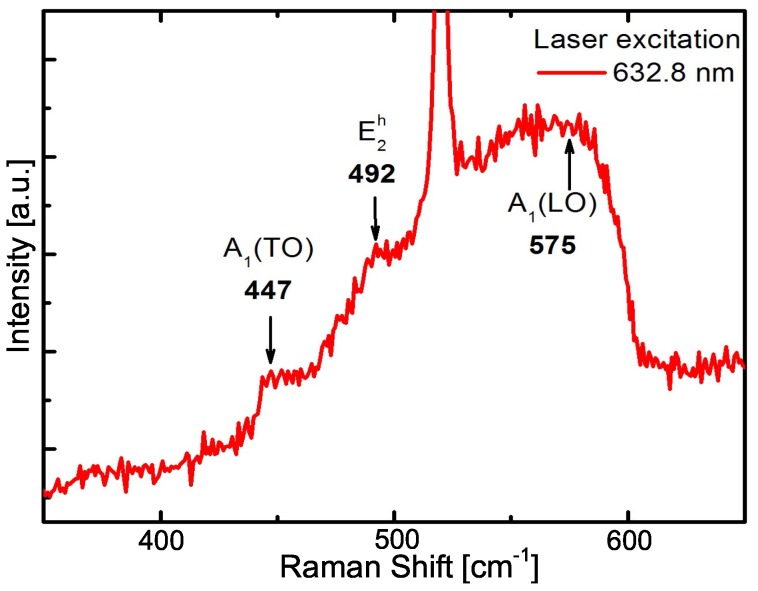
Raman spectrum obtained from the InN NCs on Si(111) substrate.

The InN in the hexagonal wurtzite structure belongs to the space group $C_{6v}^{4}$; consequently, six Raman-active modes are allowed: 2E${}_{2}$, 2A${}_{1}$ and 2E${}_{1}$ [30]. However, in backscattering geometry with incident and scattered polarization parallel, both the E${}_{2}^{h}$ and A${}_{1}$(LO) modes are allowed [31]. Room-temperature micro-Raman spectrum from hexagonal InN NCs measured with a laser excitation of 632.8nm is displayed in Figure 5. The Raman spectrum exhibits an intense peak at $520\;{\mathrm{cm}}^{-1}$ corresponding to the Si substrate and shows a broad band between 430 and 600 cm${}^{-1}$. Despite the presence of this wide band, it was possible to distinguish Raman peaks of the InN NCs centered at around 447, 492, and $575\;{\mathrm{cm}}^{-1}$, which are assigned to the A${}_{1}$(TO), E${}_{2}^{h}$ and A${}_{1}$(LO) phonon modes, respectively. The A${}_{1}$(TO) is a forbidden phonon mode that could be explained by the main contribution to the light scattering coming from the lateral area of the NCs [32]. The peak positions of A${}_{1}$(TO) and E${}_{2}^{h}$ modes are in close agreement with modes reported for both the InN layers [33] and the InN NCs [31]. The frequency assigned to E${}_{2}^{h}$ mode, is most sensitive to strain, and therefore is evidence that the NCs are relaxed, as was previously inferred from XRD and TEM studies. The A${}_{1}$(LO) mode in the InN NCs is shifted towards lower values compared with the commonly reported value of $588\;{\mathrm{cm}}^{-1}$ [31]. This result could be attributed to size-confinement effects. It means that momentum conservation is relaxed and Raman active modes are not limited to be localized at the center of the Brillouin Zone [34].

### 3.2. Optical Characterization

**Figure 6:**
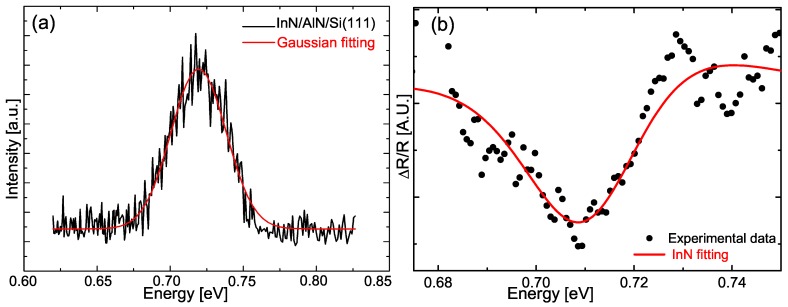
(**a**) PL spectrum measured at 20K from the InN NCs; the continuous line corresponds to the best fit to a Gaussian line shape. The PL peak is centered at 0.72eV with a FWHM of 37meV. (**b**) PR spectrum from the NCs measured at 300K, the continuous line represents the fitting by using the Equation (1).

The optical properties of the InN NCs were investigated by PL and PR spectroscopy. The PL spectrum measured at 20K of the InN NCs is shown in Figure 6a. The NCs presented a band-edge quasi-symmetrical infrared emission that is well fitted with a Gaussian curve centered at 0.72eV [35], and a full width at half maximum (FWHM) of 37meV. Additionally, PR spectroscopy was also employed to detect the optical transition of the NCs due to its high sensitivity in the measurements [36]. The PR spectrum at 300K of the NCs is presented in Figure 6b. The reflectance of the InN NCs proved hard to modulate optically; therefore, the original PR data were Fourier transform (FT) filtered. Then, the spectrum obtained was fitted using the Aspnes lineshape given by [37]:

(1) $$\begin{array}{c} \frac{\Delta R}{R}=b+Re\left(\frac{Ce^{i\theta }}{{\left(x-E_{t}+i\Gamma \right)}^{n}}\right) \end{array}$$

where $E_{t}$ is the transition energy of the NCs; and *b*, *C*, θ, and Γ are the baseline, amplitude, phase and line width, respectively. Here, n=2.5, which corresponds to a bulk-like transition due to the size of the InN NCs. The energy $E_{t}$ obtained was $E_{t}=0.712\;\mathrm{eV}$, which supports the PL results.

## 4. Conclusions

We grew InN NCs on Si(111) substrates with high crystalline quality at a low growth temperature of $400{\;}^{\circ }C$. Our results evidence that the Al thin layer covered Si(111) substrate avoided substrate nitridation and also promoted the formation of unintentional AlN when the N-plasma was ignited. We found that the process of InN nucleation at $400{\;}^{\circ }C$ started by a layer–layer (2D) growth, followed by the growth of 3D islands, generating a transition of 2D to 3D growth. We identified nanocolumns with cylindrical and conical shapes and heights between 250 and 380 nm. The diameter of the cylindrical NCs was below 50nm and for the conical NCs below of 150nm. HR-TEM analysis revealed that the InN NCs were free of structural defects and strain. With Raman spectrocopy, the A${}_{1}$(TO), E${}_{2}^{h}$, and A${}_{1}$(LO) phonon modes corresponding to the InN are visible. At a temperature of 20K, the PL measurement showed an infrared emission at 0.72eV with a FWHM of 37meV. The PR characterization was also employed to obtain the energy transition of the NCs. The transition was found at 0.712eV at the temperature of 300K. These results are useful for the development of devices with improved performance as well as to obtain compatibility of the nitrides with current microelectronic industry based on silicon.
